# Systematic Testing of Literature Reported Genetic Variation Associated with Coronary Restenosis: Results of the GENDER Study

**DOI:** 10.1371/journal.pone.0042401

**Published:** 2012-08-03

**Authors:** Jeffrey J. W. Verschuren, Stella Trompet, Iris Postmus, M. Lourdes Sampietro, Bastiaan T. Heijmans, Jeanine J. Houwing-Duistermaat, P. Eline Slagboom, J. Wouter Jukema

**Affiliations:** 1 Department of Cardiology, Leiden University Medical Center, Leiden, The Netherlands; 2 Department of Gerontology and Geriatrics, Leiden University Medical Center, Leiden, The Netherlands; 3 Department Human Genetics, Leiden University Medical Center, Leiden, The Netherlands; 4 Molecular Epidemiology, Leiden University Medical Center, Leiden, The Netherlands; 5 Department of Medical Statistics and Bioinformatics, Leiden University Medical Center, Leiden, The Netherlands; 6 Netherlands Consortium for Healthy Ageing, Leiden, The Netherlands; 7 Interuniversity Cardiology Institute of the Netherlands (ICIN), Utrecht, The Netherlands; 8 Durrer Center for Cardiogenetic Research, Amsterdam, The Netherlands; College of Pharmacy, University of Florida, United States of America

## Abstract

**Background:**

Coronary restenosis after percutaneous coronary intervention still remains a significant problem, despite all medical advances. Unraveling the mechanisms leading to restenosis development remains challenging. Many studies have identified genetic markers associated with restenosis, but consistent replication of the reported markers is scarce. The aim of the current study was to analyze the joined effect of previously in literature reported candidate genes for restenosis in the GENetic DEterminants of Restenosis (GENDER) databank.

**Methodology/Principal Findings:**

Candidate genes were selected using a MEDLINE search including the terms ‘genetic polymorphism’ and ‘coronary restenosis’. The final set included 36 genes. Subsequently, all single nucleotide polymorphisms (SNPs) in the genomic region of these genes were analyzed in GENDER using set-based analysis in PLINK. The GENDER databank contains genotypic data of 2,571,586 SNPs of 295 cases with restenosis and 571 matched controls. The set, including all 36 literature reported genes, was, indeed, significantly associated with restenosis, p = 0.024 in the GENDER study. Subsequent analyses of the individual genes demonstrated that the observed association of the complete set was determined by 6 of the 36 genes.

**Conclusion:**

Despite overt inconsistencies in literature, with regard to individual candidate gene studies, this is the first study demonstrating that the joint effect of all these genes together, indeed, is associated with restenosis.

## Introduction

Restenosis is a complex disease for which the causative mechanisms have not yet been fully identified. Despite medical advances, restenosis still remains a significant complication after percutaneous coronary intervention (PCI).[1] Identification of risk factors and underlying mechanisms could not only be useful in risk stratification of patients, they also contribute to our understanding of this condition. In addition, these factors could provide evidence on which to base individually tailored treatment and aid in the development of novel therapeutic modalities.[2] Unraveling the mechanisms leading to restenosis development remains challenging. Genetic susceptibility is known to play a role in the individuals risk of developing this complication.[1] Many studies have focused on identification of genetic markers associated with restenosis. Over the last decades genetic research has developed from candidate gene approaches [3]–[5] to multiplex arrays [6] and finally to genome wide association studies (GWAS).[7] Genetic variation in large array of plausible candidate genes have been associated with restenosis, however, consistent replication of the reported markers is scarce.[1] Possible explanations for this lack of consistency are the small sample size of many (especially relative more dated) studies, phenotype heterogeneity and lack of proper replication cohorts.

Currently more and more GWAS are being performed, investigating many diseases, including cardiovascular diseases.[8], [9] An advantage of GWAS is the hypothesis-free approach of this method, enabling identification of new genetic loci associated with the disease of interest. With respect to restenosis, a disadvantage of the GWAS approach is that due to the complexity of the disease the effect size of individual genetic markers is likely to be small and therefore hard to detect. Moreover, the availability of (large) replication cohorts is very limited. In 2011, the first GWAS on restenosis in the GENetic DEterminants of Restenosis (GENDER) study identified a new susceptibility locus on chromosome 12.[7] The fact that this GWAS only identified this previously unknown locus does not mean that genetic variation in the previously proposed candidate genes does not affect restenosis development. It merely indicates that the influence of other individual markers is probably too small to detect in the GWAS setting. Especially for the complex traits, a more appropriate approach to interpret GWAS data is to analyze the combined effect of a single nucleotide polymorphism (SNP) set, grouped per pathway or gene region.[10] To date, investigation into a possible joined effect of multiple genetic markers for restenosis has not been performed.

The goal of the current study is to investigate whether the last decade of research on genetics of restenosis has led to a set of genes that is associated with restenosis in a set-based analysis using the available genotypic data of the GENDER databank.

## Methods

### Gene Selection

Candidate genes previously associated with restenosis were selected after a search of literature of papers published up to November 2011. Genes were identified searching MEDLINE using keywords as ‘genetic polymorphism’, ‘candidate gene’, ‘restenosis’ and ‘percutaneous coronary intervention’. Selection criteria included a sample size of >250 patients and the observation of a significant association of a SNP with restenosis. The final set included 36 genes. All available SNPs from the GENDER GWAS databank within a 10-Kb window around these genes were analyzed.

### Study Population

The design of GENDER and the genome-wide association study (GWAS), which has been performed in a subset of this study population, have both been described previously.[7], [11] In brief, GENDER included 3,104 consecutive unrelated symptomatic patients treated successfully by PCI for angina. The study protocol conforms to the Declaration of Helsinki and was approved by the ethics committees of each participating institution. Written informed consent was obtained from each participant before the PCI procedure. During a follow-up period of 9 months, the endpoint clinical restenosis, defined as renewed symptoms requiring target vessel revascularization (TVR) either by repeated PCI or CABG, by death from cardiac causes or myocardial infarction not attributable to another coronary event than the target vessel, was recorded. During follow-up, 346 patients developed clinical restenosis. Blood samples were collected at the index procedure for DNA isolation. The GWAS was performed in 325 cases of restenosis and 630 controls matched by gender, age, and some possible confounding clinical variables for restenosis in the GENDER study such as total occlusion, diabetes, current smoking and residual stenosis. Genotyping was performed using the Illumina Human 610-Quad Beadchips following the manufacturer’s instructions. After genotyping, samples and genetic markers were subjected to a stringent quality control protocol. The final dataset consisted of 866 individuals (295 cases, 571 controls) and 556,099 SNPs that passed all quality control criteria, together covering 89% of the common genetic variation in the European population.[7], [12] Imputation was performed with MACH software based on the HapMap II release 22 CEU build 36 using the default settings.[13] This program infers missing genotypes based on the known genotypic data of the samples together with haplotypes from a reference population provided by HapMap taken into account the degree of linkage disequilibrium (LD). After subsequent quality control, we excluded SNPs for further analyses with a call rate lower than 95% (n = 3335) or with a significant deviation from Hardy–Weinberg equilibrium (HWE) in controls (P<0.00001) (n = 79). The final GENDER Biobank dataset consisted of 866 (295 cases, 571 controls) individuals and 2,571,586 SNPs.

### Statistical Analysis

The statistical analyses were performed using the set-based test of PLINK v1.07.[14] During this test, first a single SNP analysis of all SNPs within the set is performed. Subsequently a mean SNP statistic is calculated from the single SNP statistics of a maximum amount of independent SNPs below a certain p-value threshold. If SNPs are not independent and the LD (expressed in R^2^) is above a certain threshold, the SNP with the lowest p-value in the single SNP analysis is selected. This analysis is repeated in a certain amount of permutations of the phenotype. An empirical p-value for the SNP set is computed by calculating the number of times the test statistic of the simulated SNP sets exceeds that of the original SNP set. For the analysis of this study, the parameters were set to p-value threshold <0.05, R^2^ threshold <0.1, maximum number of SNPs  = 5 and 10,000 permutations.

Initially, the set including all 36 genes is tested as a whole for the association with restenosis. Subsequent analysis of the individual genes will be justified only when the complete set is significantly associated with the endpoint.

## Results

Patient characteristics are presented in Table 1. No significant differences were found between cases and controls regarding the known risk factors for restenosis (age, diabetes, smoking, stenting and previous restenosis). Hypertension and multivessel disease were more common in the cases compared to the controls.

**Table 1:** Demographic, clinical and lesion characteristics of the study population.

	Cases (n = 295)	Controls (n = 571)	p-value
Age (years)	62.8±10.6	62.4±10.9	0.59
BMI (kg.m^−2^)	26.7±3.6	27.1±3.7	0.20
Male sex	213 (72)	421 (74)	0.63
Diabetes	58 (20)	119 (21)	0.68
Hypercholesterolemia	179 (61)	341 (60)	0.79
Hypertension	138 (47)	211 (37)	0.005
Current smoker	68 (23)	148 (26)	0.36
Family history of MI	117 (40)	210 (37)	0.41
Previous MI	119 (40)	246 (43)	0.44
Stable angina	188 (64)	400 (68)	0.06
Multivessel disease	155 (53)	248 (43)	0.01
Restenotic lesion	23 (8)	48 (8)	0.76
Total occlusion	57 (19)	97 (17)	0.40
Type C lesion	95 (38)	154 (27)	0.11
Stenting	199 (68)	385 (67)	0.99

Values were given as n (%) or mean ± SD. Patients using anti-diabetic medication or insulin at study entry were considered to be diabetics. Hypertension was defined as a blood pressure of either above 160 mmHg systolic or 90 mmHg diastolic. Hypercholesterolaemia was defined as total cholesterol concentrations of above 5 mmol/L. BMI: body mass index, MI: myocardial infarction. P-values are determined by Pearsons Chi-Square (discrete variables) or unpaired 2-sided t-test (continuous variables).

**Figure 1:**
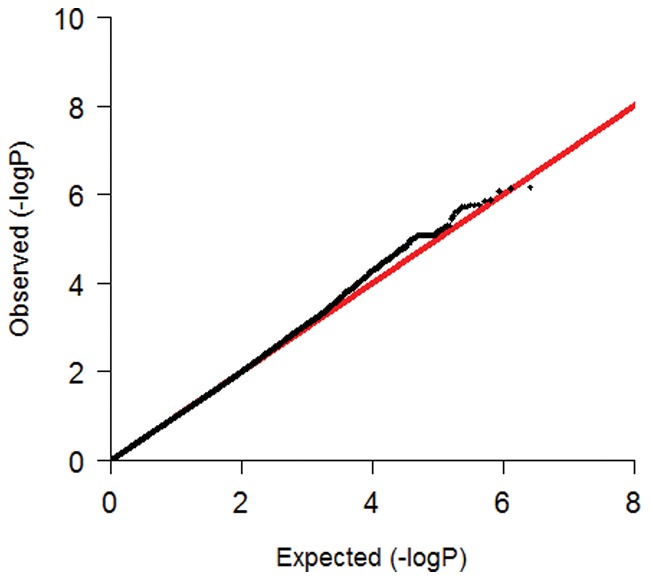
Q-Q plot for the GWAS after imputation on clinical restenosis in the GENDER study population. Lambda  = 1.027.

In Figure 1 the QQ-plot of the GENDER GWAS after imputation is shown, demonstrating that no genomic inflation has occurred in this analysis (lambda  = 1.027). The complete set of 36 genes, previously associated with restenosis in literature, contained 2,581 SNPs. A detailed description of the individual studies and candidate genes can be found in Table 2. The largest gene was chemokine (C-X3-C motif) receptor 1 (CX3CR1) of 316.54 kb, contributing 384 SNPs (14.8%), and glutathione peroxidase 1 (GPX1) was with 1.18 kb the smallest gene, only contributing 8 SNPs (0.3%). Analysis of the complete set using the set-based test demonstrated a significant association with clinical restenosis, with an empirical p-value of 0.024.

**Table 2:** Candidate genes and the studies that reported their association with restenosis.

Candidate gene			Literature based study characteristics and results					
Gene	Entrez nr	Location	Study size	% of cases	Follow-up (mo)	Top SNP	Effect size (95% CI)^a^	Ref
adrenergic beta-2-receptor (ADRB2)	154	5q31–q32	3104	9.8	9	rs1042713	HR 1.33 (1.06–1.68)	[6]
advanced glycosylation end product-specific receptor (AGER)	177	6p21.3	267	UK	6–9	rs1800624	↓	[24]
			297	25.9	6	rs2070600	NS	[25]
angiotensin II receptor, type 1 (AGTR1)	184	3q24	272	29.8	6	rs5186	NS	[26]
			3104	9.8	9	rs5186	OR 1.85 (1.28–2.66)	[27]
Butyrylcholinesterase (BCHE)	590	3q26.1–q26.2	461	23.2	6	rs1803274	OR 5.5 (1.6–21.4)	[28]
chemokine (C–C motif) ligand 11 (CCL11)	6356	17q21.1–q21.2	3104	9.8	9	rs4795895	HR 0.73 (0.58–0.93)	[6]
CD14	929	5q31.1	129	24	6	rs2569190	RR 3.8 (1.2–11.6)	[29]
			3104	9.8	9	rs2569190	HR 0.74 (0.55–0.99)	[6]
cyclin-dependent kinase inhibitor 1B (p27, Kip1) (CDKN1B)	1027	12p13.1-p12	433	11.3	12	rs34330	NS	[30]
			2309	8.8	9	rs36448499	HR 0.61 (0.40–0.93)	[31]
collagen, type III, alpha 1 (Col3A1)	1281	2q31	527	9.1	6	rs1800255	OR 4.2 (1.4–11.2)	[32]
colony stimulating factor 2 (CSF2)	1437	5q31.1	3104	9.8	9	rs25882	HR 0.76 (0.61–0.94)	[6]
chemokine (C-X3-C motif) receptor 1 (CX3CR1)	1524	3p21.3	365	25.5	6	rs3732379	OR 2.4 (1.3–4.2)	[33]
cytochrome b-245, alpha polypeptide (CYBA)	1535	16q24	730	35.8	6	rs4673	OR 0.5 (0.3–0.8)	[34]
cytochrome P450, family 2, subfamily C, polypeptide 19 (CYP2C19)	1557	10q24	928	19.1	12	rs12248560	↓	[35]
fibrinogen beta chain (FGB)	2244	4q28	527	9.1	6	rs1800790	OR 2.7 (1.2–6.2)	[32]
			2257	8.8	9	rs1800790	NS	[36]
coagulation factor V (F5)	2153	1q23	3104	9.8	9	rs6025	HR 0.40 (0.19–0.85)	[37]
glutathione peroxidase 1 (GPX1)	2876	3p21.3	461	23.2	6	rs1050450	OR 2.1 (1.2–3.8)	[28]
interleukin 10 (IL10)	3586	1q31–q32	162	39.5	UK	rs1800871	HR 0.39 (0.16–0.94)	[38]
			1850	17.6	12		NS	[39]
			3104	9.8	9	rs3024498	HR 2.0 (1.4–2.8)	[40]
interleukin 1 receptor antagonist (IL1RN)	3557	2q14.2	183	46.4	12	VNTR	HR 5.24 (1.63–16.81)	[41]
			779	43.9	6	VNTR	NS	[42]
			1850	20.3	12	rs419598	OR 0.73 (0.58–0.92)	[3]
insulin receptor (INSR)	3643	19p13.3–p13.2	461	23.2	6	7,067,365C>A	OR 1.9 (1.2–3.1)	[28]
integrin, beta 2 (ITGB2)	3689	21q22.3	1207	21.2	12	rs235326	OR 0.71 (0.55–0.92)	[4]
lipoprotein lipase (LPL)	4023	8p22	3104	9.8	9	rs328	OR 0.62 (0.44–0.86)	[43]
matrix metallopeptidase 12 (MMP12)	4321	11q22.3	527	9.1	6	rs2276109	OR 3.9 (1.0–12.4)	[32]
matrix metallopeptidase 9 (MMP9)	4318	20q11.2–q13.1	461	23.2	6	rs2664538	OR 2.0 (1.0–3.9)	[28]
methylenetetrahydrofolate reductase (NAD(P)H) (MTHFR)	4524	1p36.3	260	36.9	6	rs1801133	OR 3.58 (1.51–8.46)	[44]
			800	18.9	12	rs1801133	NS	[45]
nitric oxide synthase 3 (NOS3)	4846	7q36	205	29.3	6	rs2070744	OR 2.06 (1.08–3.94)	[46]
			901	10.2	9	rs1799983	HR 1.67 (1.09–2.54)	[47]
			1556	20.8	12	rs1799983	NS	[48]
purinergic receptor P2Y, G-protein coupled, 12 (P2RY12)	64805	3q24–q25	2062	8.4	9	Haplotype of 5 SNPs	HR 1.6 (1.2–2.0)	[49]
serpin peptidase inhibitor, clade E, member 1 (SERPINE1)	5054	7q21.3–q22	1850	20.3	12	rs1799899	NS	[50]
			3104	9.8	9	rs1799899	HR 1.26 (1.07–1.49)	[37]
K(lysine) acetyltransferase 2B (KAT2B, PCAF)	8850	3p24	3104	9.8	9	rs2948080	HR 0.80 (0.67–0.97)	[51]
peroxisome proliferator-activated receptor gamma (PPARG)	5468	3p25	565	28.7	6	rs3856806	↓	[52]
			935	18.3	12	rs3856806	NS	[53]
c-ros oncogene 1, receptor tyrosine kinase (ROS1)	6098	6q22	461	23.2	6	rs529038	HR 1.8 (1.1–2.8)	[28]
thrombomodulin (THBD)	7056	20p11.2	730	35.8	6	rs1042579	OR 2.1 (1.3–3.53)	[34]
thrombospondin 4 (THBS4)	7060	5q13	628	UK	6–10	rs1866389	OR 2.67 (1.04–6.80)	[54]
thrombopoietin (THPO)	7066	3q27	527	9.1	6	rs6141	OR 2.4 (1.1–5.3)	[32]
tumor necrosis factor (TNF)	7124	6p21.3	1850	17.6	12	rs1800629	NS	[39]
			3104	9.8	9	rs361525	HR 0.60 (0.37–0.98)	[5]
tumor protein p53 (TP53)	7157	17p13.1	132	0	UK	rs1042522	↑	[55]
			433	11.3	12	rs1042522	NS	[30]
			779	43.9	6	Haplotype of 3 SNPs	OR 0.58 (0.40–0.83)	[56]
uncoupling protein 3 (UCP3)	7352	11q13.4	527	9.1	6	rs1800849	OR 5.2 (1.9–13.0)	[32]
vitamin D receptor (VDR)	7421	12q13.11	3104	9.8	9	Haplotype of rs11568820 and rs4516035	HR 0.72 (0.57–0.93)	[57]

a The direction of the association between genetic variation and the risk of restenosis, when effect size is not available;↓ protective effect, ↑ deleterious effect. Entrez nr; unique gene ID number used in NCBI database. Abbreviations: UK, unknown; NS, not significant; OR, odds ratio; HR, hazard ratio; RR, relative risk; Ref, reference.

To determine which genes are mainly responsible for this association we subsequently investigated the association of the individual gene based sets. Six of the 36 genes were demonstrated to have an empirical p-value below 0.05 (Table 3). In order of descending p-values the associated genes are; angiotensin II receptor type 1 (AGTR, p = 0.028), glutathione peroxidase 1 (GPX1, p = 0.025), K(lysine) acetyltransferase 2B (KAT2B, also known as PCAF, p = 0.023), matrix metallopeptidase 12 (MMP12, p = 0.019), fibrinogen beta chain (FGB, p = 0.013) and vitamin D receptor (VDR, p = 0.012). Detailed information on the individual SNPs in these genes is depicted in Table 4. The SNP with the lowest individual p-value was rs11574027 in the VDR gene, p = 1.4E-04. In the complete GWAS analysis, which has been published in 2011 [7], this SNP ranked 116^th^. The strongest association in that analysis was found with a SNP in an intergenic region on chromosome 12, p = 1.0E-06.

Logistic regression models with and without the 11 SNPs described in Table 4 demonstrated that together these SNPs explained 9.0% (R Square improved from 0.008 to 0.098) of the occurrence of clinical restenosis in this cohort.

**Table 3:** Results of individual gene set-based analysis of genes previously associated with restenosis.

Gene	Chr	Start (bp)	End (bp)	Size (kb)	SNPs	Sign. SNPs	Indep. SNPs	P-value
ADRB2	5	148 186 349	148 188 381	2.03	32	8	2	0.088
AGER	6	32 256 724	32 260 001	3.28	37	1	1	0.228
AGTR1	3	149 898 348	149 943 480	45.13	100	5	1	**0.028**
BCHE	3	166 973 387	167 037 944	64.56	101	8	2	0.314
CCL11	17	29 636 800	29 639 312	2.51	18	0	0	1.000
CD14	5	139 991 501	139 993 439	1.94	22	4	2	1.000
CDKN1B	12	12 761 576	12 766 569	4.99	13	0	0	1.000
Col3A1	2	189 547 344	189 585 717	38.37	97	2	2	0.649
CSF2	5	131 437 384	131 439 757	2.37	28	0	0	0.965
CX3CR1	3	39 279 990	39 596 531	316.54	384	3	1	0.358
CYBA	16	87 237 199	87 244 958	7.76	14	1	1	0.182
CYP2C19	10	96 512 453	96 602 660	90.21	43	1	1	1.000
FGB	4	155 703 596	155 711 686	8.09	25	2	1	**0.013**
F5	1	167 747 816	167 822 393	74.58	200	1	1	1.000
GPX1	3	49 369 615	49 370 795	1.18	8	1	1	**0.024**
IL10	1	205 007 571	205 012 462	4.89	30	5	1	**0.053**
IL1RN	2	113 601 609	113 608 063	6.45	62	0	0	0.991
INSR	19	7 063 266	7 245 011	181.75	172	20	5	0.263
ITGB2	21	45 130 299	45 165 303	35.00	57	6	4	0.663
LPL	8	19 841 058	19 869 049	27.99	75	14	5	1.000
MMP12	11	102 238 675	102 250 922	12.25	36	3	3	**0.019**
MMP9	20	44 070 954	44 078 606	7.65	23	10	3	0.067
MTHFR	1	11 768 374	11 788 702	20.33	61	1	1	1.000
NOS3	7	150 319 080	150 342 608	23.53	20	0	0	0.987
P2RY12	3	152 538 066	152 585 234	47.17	121	0	0	1.000
SERPINE1	7	100 556 303	100 558 421	2.12	27	0	0	0.863
KAT2B	3	20 056 528	20 170 898	114.37	144	19	4	**0.023**
PPARG	3	12 304 349	12 450 854	146.51	144	14	5	1.000
ROS1	6	117 716 223	117 853 711	137.49	206	1	1	0.631
THBD	20	22 974 271	22 978 301	4.03	22	0	0	1.000
THBS4	5	79 366 747	79 414 861	48.11	61	3	2	0.292
THPO	3	185 572 467	185 578 626	6.16	16	1	1	0.165
TNF	6	31 651 329	31 654 089	2.76	41	2	2	0.370
TP53	17	7 512 445	7 531 642	19.20	17	1	1	0.120
UCP3	11	73 388 958	73 397 778	8.82	34	1	1	0.183
VDR	12	46 521 589	46 585 081	63.49	93	2	2	**0.012**

Chromosome and genomic region based on HapMap Rel 28 Phase II+III. P-value based on permutation (10,000). Abbreviations: SNPs, number of SNPs in genomic region including 10 kb window; Sign.SNPs, number of SNPs with p<0.05; Indep.SNPs, number of significant and independent SNPs, considering threshold of R^2^<0.1.

**Table 4:** Significantly associated SNPs of the 6 top genes.

						MAF					Imputation
Gene	SNP	Chr	bp	Function	Alleles	case	control	OR	p-value	Origin	quality
AGTR1	rs5182	3	149942085	Exon, synonymous	T/C	0.43	0.50	0.75	0.0040	Genotyped	–
FGB	rs1044291	4	155712802	3′UTR	T/C	0.38	0.30	1.40	0.0028	Imputed	0.970
GPX1	rs8179164	3	49372288	Promoter	A/T	0.02	0.04	0.42	0.0077	Imputed	0.993
MMP12	rs12808148	11	102238373	Downstream	C/T	0.16	0.09	1.82	0.00021	Imputed	0.953
	rs17099726	11	102257062	Promoter	G/T	0.03	0.06	0.54	0.032	Imputed	0.957
KAT2B	rs6776870	3	20126544	Intron	G/C	0.14	0.21	0.62	0.00064	Imputed	0.999
	rs2929404	3	20069570	Intron	T/C	0.21	0.15	1.49	0.0026	Imputed	0.981
	rs17796904	3	20096353	Intron	T/C	0.16	0.12	1.43	0.012	Genotyped	–
	rs4858767	3	20141941	Intron	G/C	0.29	0.34	0.79	0.037	Imputed	0.994
VDR	rs11574027	12	46573640	Intron	T/G	0.03	0.007	4.19	0.00014	Genotyped	–
	rs11574077	12	46539194	Intron	G/A	0.07	0.04	1.60	0.029	Genotyped	–

SNP, single nucleotide polymorphism; Chr, chromosome; bp, base pair; MAF, minor allele frequency in control group; OR, odds ratio. The imputation quality indicates the average posterior probability for the most likely genotype generated by MACH, ranging from 0–1.

As a final analysis we removed the 6 significantly associated genes from the complete set. Subsequent analysis of the subset of the other 30 genes did not demonstrate a remaining joined effect, p = 0.65 after 10,000 permutations.

## Discussion

With this study we aimed at clarifying the ambiguities regarding genetic predisposition for developing restenosis after PCI. We show that the joined effect of the complete spectrum of candidate genes, so far proposed to be involved in the restenotic process, results in a significant association with restenosis. This association is determined by six individual genes. Analyzing a subset containing the 30 genes not associated with the endpoint on an individual basis, did not show a remaining joined effect, making the involvement of genetic variation in these genes on restenosis development more unlikely.

The six associated genes span a wide range of different functions underlining the complexity of the disease. When examining the biological pathways with involvement of these genes, only the VDR and KAT2B genes share a common pathway. The genes are both involved in the Vitamin D receptor pathway described by BioCarta.[15] This pathway mainly involves the transcriptional regulating capacities of this receptor and is involved in controlling cellular growth, differentiation and apoptosis. Since these processes are all thought to be important contributors to the restenotic process, this indeed is a plausible pathway to be involved in restenosis development.[1].

The rationale of set-based analysis is to overcome the marginally weak effect of single SNPs by analyzing a set of SNPs, since these SNPs could jointly have strong genetic effects. Most studies utilizing the candidate gene approach analyzed only one or at most a few SNPs within the gene of interest. The likelihood that exactly those SNPs are the causal or associated SNPs is of course small. A broader approach, like this set-based analysis, is therefore more likely to detect an associated gene by combining multiple SNPs with a possible marginal individual effect.[16], [17] For the current study we used the PLINK software [14], although multiple statistical programs are available for this type of analysis. Gui et al. compared 7 tests analyzing the WTCCC Crohn’s Disease dataset.[18] One of their overall conclusions was that the set-based test in PLINK was the most powerful algorithm. Another study, applying PLINK set-based test, Global test, GRASS and SNP ratio test, for the analysis of three pathways regarding human longevity observed similar results with the different tests.[19].

For the current study we analyzed the data using a threshold of linkage disequilibrium defined by R^2^≥0.1. The standard setting in PLINK is a R^2^ of 0.5. In our opinion this threshold is too high for the intended analysis for this study. A higher threshold will include more SNPs in higher LD, which would be unfavorable, since we were interested in independent loci contributing to the risk of restenosis. By decreasing this threshold, only SNPs were selected that had a R^2^ below 0.1, and thus independent of each other.

Although hypertension and multivessel disease were more frequent in cases compared to controls we decided not to correct for these variables. In the complete GENDER population these variables were not independent predictors for restenosis development [11], so the differences in the current subpopulation likely resulted by chance during the selection process. Also, other studies provide no convincing data that hypertension is related to restenosis [1]. It is therefore unlikely that previous associations of some of the current candidates genes (VDR, FGB, AGTR1 and GPX1) with hypertension[20]–[23], have influenced our results, although this cannot be completely excluded.

A limitation of the current study could be that we analyzed imputed genotypic data, which introduces some amount of uncertainty. However, since we were interested in the combined effect of SNPs, an extensive genomic coverage was paramount for this analysis. Only analyzing the genotyped GWAS data would have resulted in the coverage of some of the smaller genes by only 1 or 2 SNPs. Therefore we decided that the more extensive genomic coverage of the imputed dataset outweighed the small introduction of possible error. A second limitation is that the analyses were only performed in the GENDER population. Availability of other populations with thorough genetic data on restenosis is however very limited. To our knowledge, the GWAS on restenosis in the GENDER population is the first, and only, examining this endpoint on a genome wide scale. Finally, the conclusions of this study are only based on genetic analyses. Functional studies should be performed to elucidate the biological consequences of these findings.

In conclusion, with these results we demonstrate that the efforts in unraveling the genetic factors influencing the risk of restenosis of the last years has resulted in a set of genes that joint together is indeed likely to be associated with restenosis, despite the overt inconsistencies of the individual studies. Confirmation of the association of these genes with the occurrence of restenosis after PCI helps our understanding of the genetic etiology of the disease. Future additional research strategies, like biological pathway analysis of GWAS data or even (exome) sequencing, might help us find the missing heritability of restenosis after PCI and increase our knowledge of the biological mechanistic background of restenosis development. This knowledge could subsequently result in identification of new treatment targets or development of novel preventive measure or risk stratification models.
